# Maturity assessment of KenyaEMR: advancing digital health solutions through continuous improvement in Kenya

**DOI:** 10.1186/s12913-026-14965-6

**Published:** 2026-06-17

**Authors:** Anisa Saleh, James M. Kariuki, Davies O. Kimanga, Lora L. Sabin, Sylvia Shangani, George Owiso, Lilly M. Nyagah, Jacob O. Odhiambo, Evans Munene, Jeremiah M. Mumo, Veronika J. Wirtz

**Affiliations:** 1 Boston University School of Public Health, Boston, MA USA; 2 US Centers for Disease Control and Prevention, Atlanta, GA USA; 3 United States Agency for International Development, Abuja, Nigeria; 4 Palladium Kenya, Nairobi, Kenya; 5 National AIDS and STIs Control Programme, Ministry of Health, Nairobi, Kenya; 6 Digital Health Agency, Ministry of Health, Nairobi, Kenya

**Keywords:** KenyaEMR, HIS SOCI toolkit, Maturity assessment, Health information system, Digital health solution, MEASURE Evaluation

## Abstract

**Background:**

Kenya Electronic Medical Records (KenyaEMR) has improved data availability for patient services and programmatic purposes in Kenya. Maturity models provide a framework to assess health information systems (HIS) capabilities across maturity levels to ensure they are effective. This study aimed to determine KenyaEMR’s current and goal maturity status, highlight gaps, and establish priorities for continuous improvement.

**Methods:**

The HIS Stages of Continuous Improvement (SOCI) Toolkit guided KenyaEMR’s maturity assessment across five HIS domains and five maturity stages: 1-emerging/ad hoc; 2-repeatable, 3-defined, 4-managed, and 5-optimized. Participants included KenyaEMR experts representing the Ministry of Health, implementing partners, donor agencies, and faith-based organizations. The consensus-building process to determine final maturity scores utilized a two-round modified Delphi process. Improvement roadmaps were developed as action plans to bridge identified gaps. The assessment occurred in August 2024.

**Results:**

Thirty-two stakeholders participated in the assessment. The overall KenyaEMR average maturity score was 3.2/5. Across the five HIS domains, the highest maturity scoring domain was standards and interoperability (4.4/5) and the lowest was management and workforce (2.3/5). The other domains maturity scores include leadership and governance (2.8/5), Information and Communication Technology (ICT) infrastructure (2.9/5), data quality and use (3.7/5). Among the 39 subcomponents, 7/39 (17.9%) scored at the 5-optimized maturity stage, of which six included data exchange standards, terminology management, master facility list, unique person identity management, person data exchange, and aggregated data exchange under the standards and interoperability domain. In comparison, 6/39 (15.4%) subcomponents scored lowest at the 2-repeatable level, with three (HR policy, HIS financing plan, and resource mobilization) under the management and workforce domain. The overall goal status maturity score was 4.6/5, with all subcomponents expected to reach 4-managed or 5-optimized levels by 2027.

**Conclusions:**

KenyaEMR’s overall maturity level was 3.2/5. The workforce and management domain will require attention so that national-level investments in the HIS workforce and an increase in local funding sources can decrease the high donor funding dependency by the Kenya Ministry of Health. Conducting routine assessments coupled with the upcoming promising commitments from the local government to increase digital health investments will benefit continued support for KenyaEMR as it evolves.

**Supplementary Information:**

The online version contains supplementary material available at 10.1186/s12913-026-14965-6.

## Background

Health information systems (HIS) are a cross-cutting element of the World Health Organization (WHO) health systems framework and play a critical role in providing high-quality data for planning, management, health program evaluation, and patient care services [1]. In recent years, the term digital health solution has started to be used in the field. It is synonymously used with HIS as the field is rapidly evolving [2, 3]. The availability of quality data for decision-making is vital for the health system’s ongoing improvements and for better informing policy decisions [4, 5].

Kenya’s HIS ecosystem consists of several digital health solutions. Expanding in recent years, Kenya has encouraged the implementation of new electronic health projects [6]. Electronic medical records (EMRs) supporting patient care were among the first digital health solutions implemented in Kenya focusing on human immunodeficiency virus (HIV) [7, 8]. Kenya’s Ministry of Health (MoH) oversees EMRs to ensure appropriate implementation, maintenance, and patient safety within the broader digital health landscape [7, 9]. More recently, a Digital Health Agency (DHA) has been established through an act of parliament (Digital Health Act, 2023) to oversee digital health implementation [10]. In this new legislative regime, the Kenya Ministry of Health retained its role as the main policy making entity.

Over the years, Kenya has adopted a series of digital health policies and guidelines that include the National HIS Policy (2009) [11], and EMR-focused guidance via the Standards and Guidelines for Electronic Medical Records (2010) [12]. The latter encompass international standards when defining the design and use of EMRs and was updated to Standards and Guidelines for Primary Health Care Electronic Medical Records in Kenya (2014) [13]. Additionally, the MoH published the Kenya National eHealth Strategy (2011–2017) [6, 14], the Kenya National eHealth Policy (2016–2030) [15], and the Digital Health Act (2023) [10], which established the DHA to regulate digital health in Kenya and lead development of a comprehensive integrated health management information system envisioned in the Health Act, 2017 [10]. Moreover, the MoH developed the Digital Health Superhighway (2023) blueprint that consists of National Shared Registries and an Enterprise Service Bus that will enable standards-based interoperability in the health enterprise through a robust national health information exchange [16].

Kenya Electronic Medical Records (KenyaEMR) is a distribution of the open-source Open Medical Records System (OpenMRS) platform. OpenMRS is recognized as a digital Global Good that can be customized locally to meet a country’s particular needs [17, 18]. Kenya was the first country to implement OpenMRS and the system has since been implemented at health facilities providing health services including HIV care across many low- and middle-income countries (LMICs), including sub-Saharan Africa (SSA) [17, 19]. KenyaEMR was launched in 2011/12 across public, private, non-governmental, and faith-based health facilities in Kenya, creating a foundation for providing HIV and tuberculosis care services by different health providers [8, 20]. As such, KenyaEMR is one of the longest-implemented electronic medical records (EMR) systems in Kenya and one of the systems of choice for national rollout [20]. It has managed comprehensive health services in over 2,400 health facilities nationwide, covering over 90% of patients on antiretroviral therapy (ART) as of December 2024 [17, 20–23].

Understanding the growth of digital health solutions and the changes such a system undergoes over time is critical to the knowledge of technology adaptation, which in turn has the potential to facilitate future health-related technological advances. Maturity models or stages of growth, is based on the notion that processes, capacity and capabilities undergo a series of progressing stages as they advance to an optimal level [24, 25]. To ensure that a digital health solution evolves to be well-functioning, it is essential to know the system’s state and understand what will be needed to ensure it can function and perform optimally [26]. Maturity models provide a framework for assessing the current status of a digital health solution and identifying its capabilities. This facilitates development of action plans to advance the system’s performance [25].

The MEASURE Evaluation HIS Stages of Continuous Improvement (HIS SOCI) Toolkit is a maturity framework that focuses on the importance of HIS progression towards a mature HIS, where quality data is available and used to better meet a county’s health goals [26, 27]. This toolkit contains thirty-nine subcomponents grouped in 13 components, covering five key HIS domains: (1) Leadership and Governance, (2) Management and Workforce, (3) Information and Communication Technologies (ICT) Infrastructure, (4) Standards and Interoperability, and (5) Data Quality and Use) [26], that measures maturity across five stages (Table 1). The maturity levels for each domain are evaluated on a 1–5 scale, where higher values represent advanced or fully matured processes, capacities, and capabilities.

**Table 1:** HIS stages of continuous measurement scale and description.

Stage	Description
1-Emerging/ ad hoc	Formal processes, capabilities, experience, or understanding of HIS issues/activities are limited or emerging.
	Formal processes are not documented, and functional capabilities are at the development stage.
	Success depends on individual effort.
2-Repeatable	Basic processes are in place, based on previous activities or existing and accessible policies.
	The need for standardized processes and automated functional capabilities is known.
	There are efforts to document current processes.
3-Defined	There are approved documented processes and guidelines tailored to HIS projects or activities.
	There is increased collaboration and knowledge sharing.
	Innovative methods and tools can be implemented and used to extend functional capabilities.
4-Managed	Activities are under control using established processes.
	Requirements/goals have been developed and a feedback process is in place to ensure that they are met.
	Detailed measures for processes and products are being collected.
5-Optimized	Best practices are being applied, and the system is capable of learning and adapting.
	The system uses experiences and feedback to correct problems and continuously improve processes and capabilities.
	Future challenges are anticipated, and a plan is in place to address them through innovation and new technology.
	Processes are in place to ensure review and incorporation of relevant innovation.

Source: MEASURE evaluation. User guide, HIS SOCI toolkit. 2019 [26]

The toolkit is adaptable for different settings, including LMICs, and has been validated and used in similar regional settings in Ethiopia [5] and Uganda [26]. Only one maturity assessment focused on interoperability was conducted in Kenya in 2017 [28]. No maturity assessments have been published on the progression of a single HIS. Conducting digital health solutions maturity assessments is imperative to strengthening use of such systems in healthcare provision, including emphasizing the role leaders play in prioritizing and utilizing the results of assessments [29]. This study aimed to determine KenyaEMR’s current and goal/desired maturity levels status using the HIS SOCI toolkit, identify gaps, and establish priorities for continuous improvement to meet health information needs more effectively.

## Methods

**Fig. 1:**
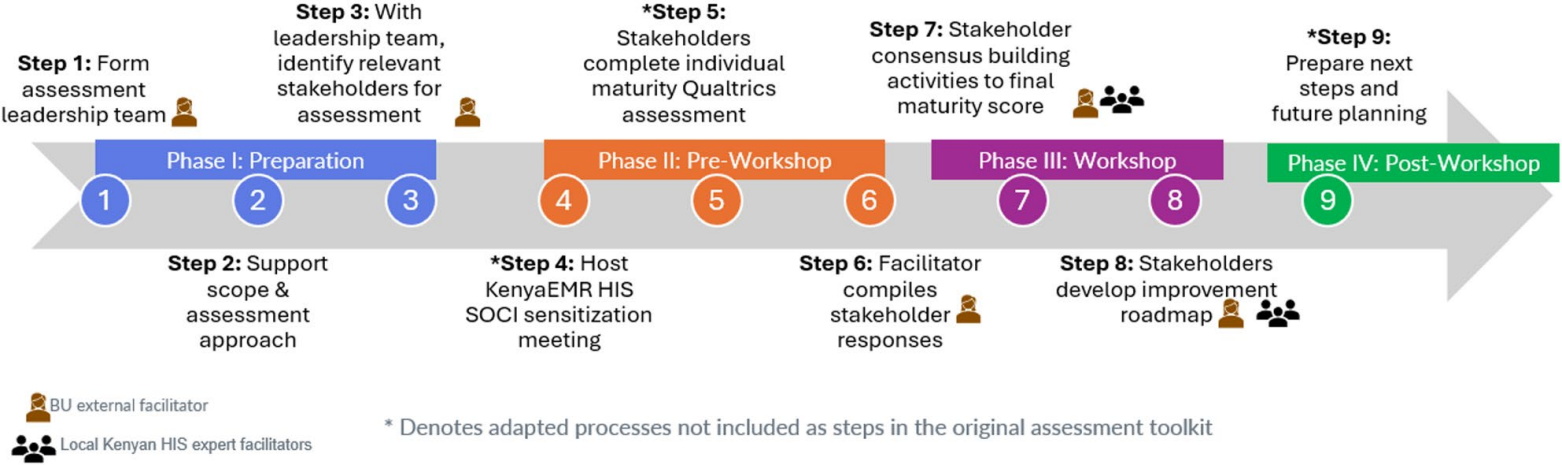
Overview of HIS SOCI maturity assessment process, adapted for KenyaEMR assessment

The assessment consisted of four sequential phases: preparation, pre-workshop, workshop and post workshop as illustrated in Fig. 1 and described below:

### Phase I: assessment preparation

In *Step 1*, a leadership team was formed to advise, steer and support the maturity assessment. The leadership team eligibility criteria included those in leadership positions, close working knowledge of KenyaEMR, and representing different organizations and perspectives from the public, private, and donor agencies. The leadership team consisted of five members that included HIS leaders from the Kenyan MoH’s Directorate of Digital Health Policy, Informatics, Policy and Research (DHIPR) and the Division of National AIDS and STI Control Programme (NASCOP), the U.S. Centers for Disease Control and Prevention (CDC) Kenya Office, Palladium Kenya, and an external facilitator from Boston University School of Public Health (BUSPH). In *step 2*, the maturity assessment scope focused on KenyaEMR, one of the longest-implemented EMR operating in over 2,400 health facilities and supported for national rollout [17, 20–23]. A hybrid approach using a self-administered (local HIS leadership team) and externally administered (BUSPH facilitator) approach was utilized to reduce bias. The MEASURE Evaluation HIS SOCI Toolkit [26], which has been previously published and made publicly available by MEASURE Evaluation, was adapted to assess KenyaEMR. This entailed adaptation of the maturity stages measurement scale definition specific to KenyaEMR to capture the progression across all thirty-nine subcomponents and their respective five stages of maturity definitions. For example, the general human resource policy definition was adapted to apply specifically to HR policies for KenyaEMR across the five stages of maturity (details in Additional file 1). The KenyaEMR adapted HIS SOCI measurement scale was converted into a Qualtrics survey platform for ease of conducting the assessment. KenyaEMR’s maturity assessment was determined at two points in time: the current status which was set to the baseline assessment period (August 2024) and the goal/desired toward an optimal system was set for December 2027, aligning with the timeline for developing the new Digital Health Strategy. In *Step 3*, the leadership team identified relevant stakeholders to participate in the KenyaEMR maturity assessment. The stakeholder selection criteria included those with KenyaEMR experience, expertise in the HIS SOCI five core domains, diverse roles, and representation from different organizations such as the MoH at national and county levels, service delivery partners, and faith-based organizations.

### Phase II: pre-workshop activities

In *Step 4*, the MoH led an initial virtual sensitization meeting with participants to explain the purpose of the assessment, provide an overview of the assessment process, demonstrate the online assessment survey formatted in the Qualtrics survey platform, and inform them about the upcoming assessment consensus-building workshop. This step is an adaptation to the HIS SOCI toolkit process for the Kenyan context.

In *Step 5*, each participant was sent an online assessment survey link with instructions to complete two core domains based on their expertise (each participant was instructed to complete the assessment for domain 3 on ICT infrastructure and another domain based on expertise) and an invitation to complete more if interest and time allowed. Assigning participants to specific groups was done to reduce the burden of competing priorities and time required to complete the assessment survey and participate in a one-day consensus-building workshop (Table 2). Assignment to each core domain group considered participant diversity based on subject matter expertise and organizational affiliation. HIS domains 1 (leadership and governance) and 2 (management and workforce) were combined in one group due to participants overlapping expertise. This was designed to ensure better distribution of participants per group and for efficient time management during the workshop. Additionally, all participants were assigned domain 3 (ICT infrastructure), because (a) it contained only five subcomponents, (b) all participants had expertise in this area, and (c) it would ensure a unified experience at the workshop launch with one central group going through the consensus-building and improvement roadmap development before breakout room sessions. Thus, a total of four groups covered the five domains.

**Table 2:** Maturity assessment group assignment by core domains

Group	Corresponding HIS core domain
A	Domain 3: ICT infrastructure
B	Domain 1 (Leadership and governance) & Domain 2 (Management and workforce)
C	Domain 4: Standards and interoperability
D	Domain: Data quality and use

**Fig. 2:**
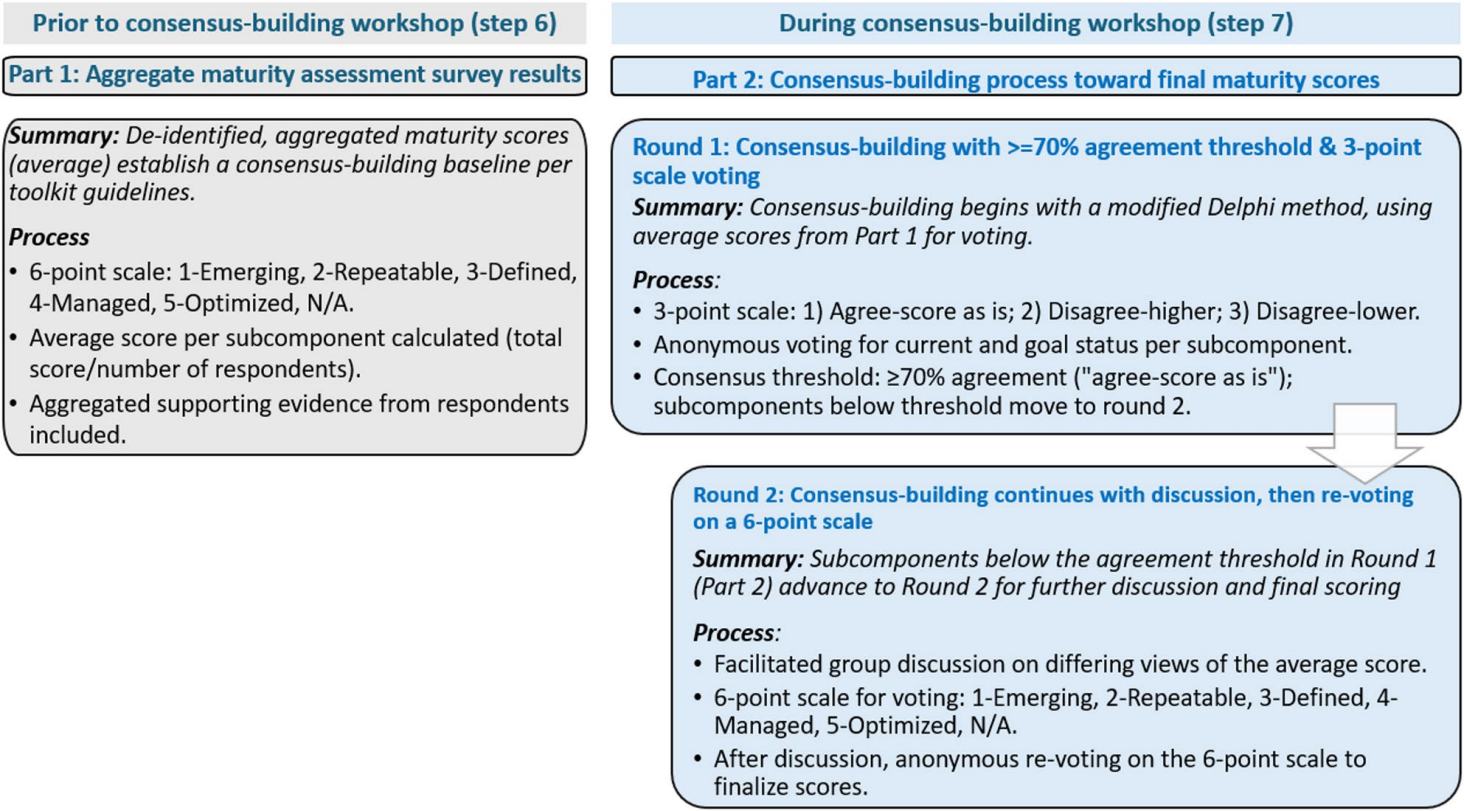
Consensus-building workflow for finalizing KenyaEMR maturity levels

In *Step 6*, individually completed survey results were aggregated by the external facilitator per domain with de-identified average scores calculated per subcomponent and any additional text with comments and evidence. Next, average scores were programmed into an anonymous poll tool, Mentimeter, in preparation for the workshop (Fig. 2).

## Phase III: maturity assessment consensus-building workshop

In *Step 7*, two rounds of anonymous polling were conducted among participants per domain group to determine consensus on final scores for the 39 subcomponents at the current (August 2024) and goal status (December 2027). The consensus-building process is informed by an adapted two-round modified Delphi process method that sets consensus at a threshold of ≥70% agreement, which aligns with the existing literature [30–32]. In round one, the average maturity score per subcomponent was displayed for the current status and goal status with a 3-point scale: ‘agree-score as is,’ ‘disagree-score should be higher,’ and ‘disagree-score should be lower.’ Consensus was determined reached if ≥70% of participants voted for ‘agree-score as is’. If this threshold was not met, the subcomponent was marked to move to round two, which entailed group discussion and then re-voting on a six-point scale: 1-emerging, 2-repeatable, 3-defined, 4-managed, 5-optimized, and N/A. After round two of voting, the average score was calculated as the final consensus score (Fig. 2). In *Step 8*, each group discussed priority items to develop an improvement roadmap with action plans to close gaps between the current and goal status and to identify resources needed. In steps 7 and 8, the facilitators led the anonymous voting process and facilitated discussions between participants to determine action items.

### Phase IV: dissemination

In the last step, dissemination involved presenting results throughout the assessment via a plenary-style presentation of action plans per domain to showcase activities highlighted and for feedback at the end of the consensus-building workshop. Additionally, preliminary assessment results were shared to the MoH and participants after the workshop.

## Results

Thirty-two experts, including strategic information advisors/leads, monitoring and evaluation officers, clinicians/health workers, HIS specialists, software developers, and health informatics officers, participated in the KenyaEMR maturity assessment, of which eighteen attended the consensus-building workshop (Table 3).

**Table 3:** Overview of organizations and participants represented in the assessment

Organization	Number of participants
Ministry of Health, national level	6
Ministry of Health, county level	5
U.S. Government (USG) agency	5
Service delivery partners and faith-based organizations	16
Total	32

The participants utilized an online assessment survey, built consensus through real-time anonymous voting per subcomponent for the current and goal status, and discussed the aggregated scores to help document action plans in an improvement roadmap. These processes enriched the discussions and efficiently completed the one-day consensus-building workshop. During consensus-building, of the thirty-nine subcomponents, twenty-three scoring instances, whether for the current status, goal status, or both, required a second round of voting following group discussion.

### Maturity levels of KenyaEMR

**Fig. 3:**
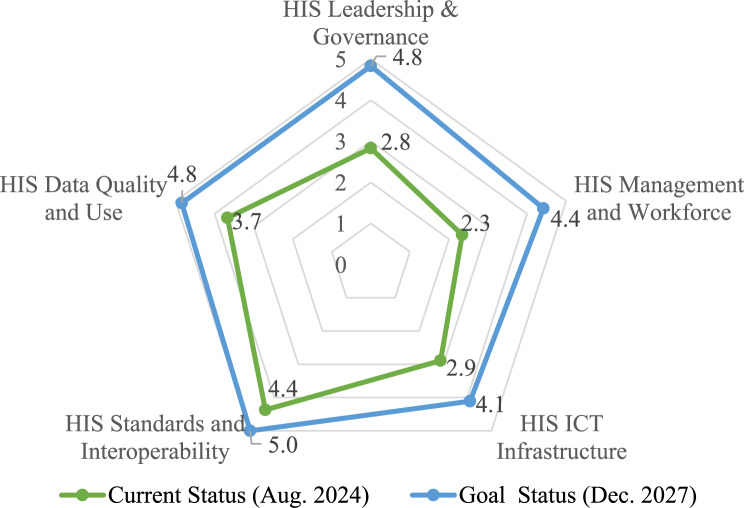
KenyaEMR maturity status by average HIS SOCI core domain score for current (Aug. 2024) and goal (Dec. 2027) status

KenyaEMR was assessed across five core domains for the five maturity levels described in the HIS SOCI measurement scale. The overall average maturity level of KenyaEMR across the core domains was at 3.2/5 for the current status and 4.6/5 for the desired goal status. Figure 3 summarizes the average maturity status score of KenyaEMR for the five core domains.

**Fig. 4:**
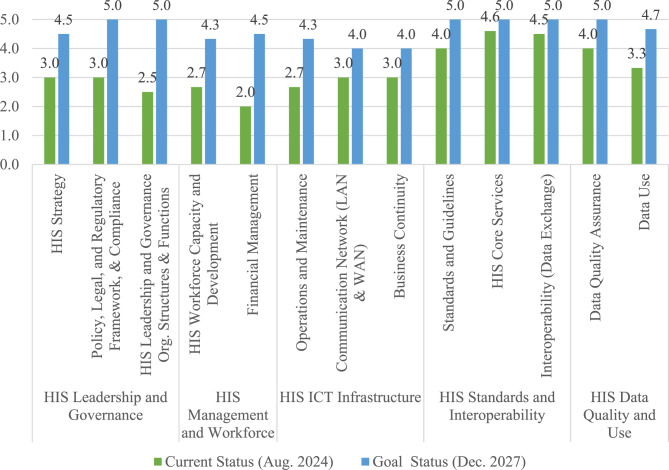
Maturity status of KenyaEMR by average HIS SOCI components score for the current status (Aug. 2024) and goal status (Dec. 2027)

The maturity level scale ranges from 1-emerging/ad hoc to 5-optimized is defined in Table 1. For the current status, three HIS core domains (leadership and governance, management and workforce, and ICT infrastructure) were between the 2-repeatable and 3-defined levels. The HIS data quality and use domain scored between 3-defined and 4-managed levels and HIS standards and interoperability scored between 4-managed and 5-optimized levels. For the goal status, the HIS standards and interoperability core domain was at the 5-optimized level, while the other four core domains were between 4-managed and 5-optimized levels. Figure 4 provides detailed scores for the maturity levels in all components within each domain. The improvement roadmap with activities to close gaps between current and goal status is in Table 4.

**Table 4:** KenyaEMR maturity improvement roadmap

		Activity	Gap Addressed	Resources needed
**Domain 1: HIS leadership and governance**				
1.		Review process of how KenyaEMR upgrade roll outs happen as part of the Digital Health Strategy – end product is that it is included in final strategy	Lack of documented process to review upgrades	COE (community of experts) meeting - time requirement
2.		Inclusion of KenyaEMR infrastructure support in national and subnational budget	KenyaEMR not included in national and subnational govt	Advocacy meeting and technical capacity to budget
**Domain 2: HIS management and workforce**				
3.		Staff establishment plan within the different cadres - getting job descriptions within partner agency to be readily available with the MoH	No central place with job descriptions, job titles, cadres, etc. regarding KenyaEMR staffing	TA from IPs on the descriptions etc. Financial resources for sub-national staff consultation
4.		Capacity building within MoH – training programmers, developers, business analyst etc. MOH allocates finance and providing paid leave for staff to grow capacity (academia, training course, etc.). Using MoH virtual learning academy for e-trainings under UoN	Gap in capacity with MoH	Time off, finance from MoH to subsidize training, willingness of staff to take trainings
**Domain 3: ICT infrastructure**				
5.		Develop a national plan to replace broken equipment for KenyaEMR	Broken and obsolete equipment are present in some places	Technical support from government. Financial support from partners/donor. PPP as another source of finance
6.		Existing business continuity plan/policy (BCP) for better dissemination to counties	BCP is not well disseminated	Using meetings with counties (virtual or in person)
7.		Routine assessment of equipment gaps regarding equipment status	Some equipment may need to be retired or obsolete since initial implementation in 2012	Counties to carry out assessments with guidance with digital health directorate
**Domain 4: HIS standards and interoperability**				
8.	Interoperability of KenyaEMR with Kenya Medical Supplies Authority Logistics Management Information System (KEMSA LMIS)		Inability to make orders automatically.	Financial Support; Technical Support; Key Stakeholders
9.	Public participation and dissemination of the Digital Health Standards and Guidelines		Public participation not yet conducted	Financial Support; Technical Support; Key Stakeholders
10.	Expand the Automated Indicator Reporting to include more datasets (i.e. commodity, pharmacy etc.) within KenyaEMR		Limited datasets in Automated Indicator Reporting.	Financial Support; Technical Support; Key Stakeholders
**Domain 5: HIS data quality and use**				
11.	Incorporating the KenyaEMR training in the preservice training		KenyaEMR training is not incorporated in preservice training.	Financial and stakeholder engagement
12.	Incorporating EMR training in the e-learning and MOH Virtual Academy.		Siloed training on programs and systems.	Finance and validation meetings
13.	Dissemination of the data use strategy to stakeholders		Lack of awareness on the existence of the data strategy	Virtual meetings
14.	Extent data from KenyaEMR is used for decision making and routine work/creating a culture of data use		Issue of evidence to show data use at different levels (HF, county, national)	Survey on data use

### Core domain 1 – HIS leadership and governance maturity levels

The current status for most (2/3) components (HIS strategy and policy, legal, and regulatory frameworks and compliance) scored at the 3-defined maturity level (Fig. 4**)**. The organization structures and functions component scored at 2.5 maturity level, attributed to the HIS organization structure and function subcomponent being at the 2-repeatable level. For the goal status, most components are scored at the 5-optimized level (2/3) or between the 4-managed and 5-optimized maturity levels (1/3). See Table 4 for the improvement roadmap (for this and all subsequent domains below).

### Core domain 2 – HIS management and workforce maturity levels

Compared to other domains, the HIS management and workforce domain scored the lowest for the current status at 2.3/5. The financial management component scored at the 2-repeatable level, which was also reflected in its two respective subcomponents, HIS financing plan and resource mobilization. The HIS workforce capacity and development component scored at 2.7, with most of its respective subcomponents at the 3-defined level (2/3). Of these, the human resource subcomponent is at the 2-repeatable level. All three components were set between the 4-managed and 5-optimized levels for the goal status.

### Core domain 3 – HIS ICT infrastructure maturity levels

The current status of two components, communication network (LAN &WAN) and business continuity, components in the HIS ICT Infrastructure domain are at the 3-defined level, while operation and maintenance component is between 2-repeatable and 3-defined at 2.7/5 (Fig. 4). The current status of four out of five subcomponents in this domain are at the defined level, except ICT business infrastructure (2.0), which is at the repeatable level. For the goal status, two components are at the 4-managed level, while one component is between the 4-managed and 5-optimized levels.

### Core domain 4 – HIS standards and interoperability maturity levels

At the current status, the HIS standards and interoperability domain had the highest overall score at 4.4/5. HIS core services and interoperability components were between 4-managed and 5-optimized levels and standards and guidelines component was) at the 4-managed level (Fig. 4). Most subcomponents scored at 4-managed or 5-optimized levels (11/12). The exception is the data set definitions subcomponent, which scored at the 3-defined level, as well-defined data sets and variables are available (including in KenyaEMR), developed, and updated in line with national guidelines; however, they are not reviewed regularly. All components and respective subcomponents are at the 5-optimized level for the goal status.

### Core domain 5 – HIS data quality and use maturity levels

At the current status, the data quality assurance component and its two respective subcomponents had a maturity score of 4. The data use component scored at 3.3 with respective subcomponents varying levels, of which most are at the 3-defined (5/11) and 4-managed (2/11) levels. The clinical decision support subcomponent scored at the optimized level of 5/5. The data use impact subcomponent scored lowest at the 2-repeatable level. All components were at the 5-optimized level or between 4-managed and 5-optimized for the goal status.

### KenyaEMR improvement roadmap

Facilitators led discussions in each group to document gaps and prioritize activities to help close gaps between the current and goal status and to identify resources needed. An improvement roadmap with action items and resources needed to close the gap was developed by the participants as summarized in Table 4.

## Discussion

This study assessed KenyaEMR’s current and goal status maturity levels, highlighted areas that require attention, and established priorities to meet health information needs more effectively across five core HIS SOCI domains. Across the five core HIS domains, the current status scored at 3.2/5, and the desired goal status scored 4.6/5. This assessment focused on a single digital health solution, KenyaEMR, compared to other HIS SOCI maturity assessments that focused on the national HIS. KenyaEMR’s overall current status maturity score is higher compared to the national HIS maturity assessments conducted in Ethiopia (2021) [5] and Uganda (2018) [26–33]. One explanation for this could be that digital health solutions in Kenya are among the longest implemented, including KenyaEMR, giving them more time for continuous improvement. They have also received substantial financial and technical support from donor agencies, implementation partners, and the Kenya MoH over the past decade.

Among the 39 subcomponents, the current status of seven subcomponents was rated at the highest maturity level (5-optimized). Six subcomponents were under the HIS standards and interoperability domain, including data exchange standards, terminology management, master facility list, unique person identity management, person data exchange, and aggregated data exchange subcomponents. Additionally, the clinical decision support subcomponent in the HIS data quality and use domain also scored at a 5-optimized maturity level as machine learning models help predict patients at different risk levels while algorithms are evaluated regularly. KenyaEMR has well-established machine learning capabilities to predict patients’ risk levels, such as treatment interruption, which better support clinicians with decision-making and targeting appropriate interventions in advance. These scores indicate that existing standards and guidelines are in place to link and share data, have national terminology services, that robust HIE exists between KenyaEMR and other systems, with unique patient identifier capabilities. These align with a prior maturity assessment of Kenya’s HIS interoperability readiness that utilized a different MEASURE Evaluation tool, where subdomains that scored the highest included the interoperability guidance documents (4-institutionalized) and the governance structure for HIS (3+, established), which are equivalent to managed and optimized levels, respectively [28].

While some domains showed strong performance, others highlighted areas for improvement. Subcomponents that scored the lowest within their domain at a 2-repeatable maturity level were mainly among the HIS management and workforce domain (3/5), followed by the HIS leadership and governance (1/6), HIS ICT infrastructure (1/5), and HIS data quality and use domains (1/11). For example, under the HIS management and workforce domain, the subcomponents on human resource policy, HIS financing plan, and resource mobilization scored at the 2-repeatable maturity level, which aligns with the findings from the HIS interoperability readiness maturity assessment conducted in Kenya [28]. The financial plans and resource mobilization for KenyaEMR and most EMRs currently come from different sources that are mainly donor-driven. However, in the future stakeholders identified these are expected to be included in the MoH’s core funding and through optimizing public-private partnerships. Resource mobilization is not synchronized as donor and partner agencies have separate priorities and planning periods, such as the U.S. Presidents Emergency Plan for Aids Relief (PEPFAR) Country Operational Plan. At the same time, the national government routinely mobilizes resources at different health levels. To make progress with financing and resource mobilization, we recommend the MoH and DHA should align resources to maximize funding from different funding sources and to improve resource mobilization and to support KenyaEMR and broader HIS initiatives in Kenya.

Additionally, based on stakeholders’ input, the current human resource policy that guides workforce planning and ensures sufficient workforce capacity and management of staff to support KenyaEMR is limited given inadequate staffing at the county and health facility levels. With county and facility levels being understaffed, the MoH relies on seconding staff from the Ministry of ICT for additional support to meet some of their needs. There is a gap in the literature to quantify the HIS workforce shortage in Kenya and other African countries. Still, challenges with inadequate staffing align with other studies that found the general human resources for health (HRH) workforce shortage to achieve UHC in Kenya had a 17.2% overall HRH availability among nurses, clinicians, nutritionists, laboratory technologists, and pharmacists [34]. Additionally, the 2015 Kenya MoH study found 1.6 health workers available for every 1,000 persons, placing it well below the WHO recommendation levels (4.6) [35], a workforce shortage challenge that is common across the African continent [36].

While the HIS management and workforce domain scored the lowest, it did not interfere with KenyaEMR’s further maturation among the system’s standards and guidelines, core services, interoperability with other systems, and data quality and use. However, when considering KenyaEMR’s future, stakeholders shared their expectation that the new DHA will have policies and structures to better manage digital health-focused staff going forward once it is fully operational. Through the improvement roadmap, stakeholders highlighted activities such as the development of a staff establishment plan among different cadres and building the capacity of the MoH using the MoH Training Academy to help bridge the existing workforce gaps. To further mitigate digital health workforce challenges, we recommend the MoH and DHA foster partnerships with universities and institutions providing pre-service clinical training, ICT, and other technical training to better prepare those who will interact with digital health solutions such as KenyaEMR.

The three other domains on data quality and use, ICT infrastructure, and leadership and governance had overall average scores of 3.6, 2.9, and 2.8 respectively. The data quality and use domain subcomponents are mainly at the 3-defined and 4-managed maturity levels, except for data use impact, which scored at 2.0/5, the lowest within the domain. This is attributed to the limited extent of KenyaEMR data used for decision-making and routine work, specifically regarding demonstrating impact, which indicates an inadequate data use culture. It may be that in its design and conceptualized use, applying data for decision making and programmatic changes to KenyaEMR was unrealistic. But it could also suggest that this is an area that matures later, when other domains have established more maturity. Certainly, advancing activities to better use data for program improvements is important and should be prioritized in future years.

Under the ICT infrastructure domain, the ICT business infrastructure subcomponent scored at 2.0/5 because of insufficient ICT infrastructure at the health facility and MoH levels to support business needs. In addition, there are no clear reporting mechanisms (e.g., no clear ways to report losses or damages by facilities and the MoH). While some facilities have dedicated ICT offices or teams, the support they receive is ad hoc and reactive, rather than having reliable, mainstreamed resources. This is similar to the operations and maintenance subdomain from the Kenya HIS interoperability maturity assessment, which is at a 2 + maturity level [28]. Moreover, one important finding is that reaching the goal status by 2027, especially on ICT infrastructure and workforce training, depends not only on the MoH and key stakeholders but also on other sectors, including the energy, ICT, and educational sectors, to ensure reliable electricity and human resources training. This interdependency is often neglected and highlights the need for investments across sectors. To mitigate these challenges, we propose that the MoH and DHA: (a) establish clear processes for reporting issues related to KenyaEMR and its products to the MoH, including equipment loss or damage, for streamlined support and (b) enhance relationships with other sectors that impact HIS infrastructure, such as sufficient reliable power/electricity, for continuous use of KenyaEMR and other digital health solutions.

For the HIS leadership and governance domain, one explanation for it scoring between the 2-repeatable and 3-defined maturity levels is that the KenyaEMR organizational structures and functions are distributed among stakeholders (e.g., implementing partners, funding agencies, and the MoH) with some support from the service delivery partners and counties. While Kenya has several policies and strategic plans in place, the roles and responsibilities of the different stakeholders supporting KenyaEMR are not clearly outlined in one place. This will be pertinent as some organizational structures transition to the new DHA. Furthermore, not all participating stakeholders were aware of new or in-progress policies regarding KenyaEMR or digital health, which led to information asymmetry among stakeholders. While this was resolved through discussion, routine dissemination of recent updates to all relevant stakeholders is key for comprehensive awareness of updates. To support progress in this domain, we recommend (a) the MoH establishes a routine process to disseminate updates on policy developments to all digital health stakeholders by leveraging existing forums (e.g., webinars, technical working group meetings, MoH website). This can reduce information asymmetry to ensure stakeholders are consistently informed, including future assessments and beyond; (b) MoH and DHA develop clear organizational roles, functions, and responsibilities across stakeholders (i.e., funding agencies, MoH, and service delivery partners) supporting KenyaEMR. A RACI matrix (Responsible, Accountable, Consulted, Informed) [37] often used in lean or project management processes can guide the efficient assigning organizational roles to monitor KenyaEMR; (c) the MoH and DHA develop a phased transition plan that outlines the expected responsibilities of the DHA in supporting KenyaEMR and other digital health services, detailing the steps needed to bridge current and goal status, align with the improvement roadmap, and set clear timelines to achieve expected outcomes. Improving communication will be an important mandate for the new DHA.

The consensus-building workshop demonstrated several key areas that required extended discussion among stakeholders. The subcomponents that did not meet the ≥70% agreement threshold for the ‘agree-score as is’ option during round one led to further discussions in round two, as some subcomponents took longer to determine consensus among stakeholders. Within the HIS leadership and governance domain, one example of subcomponents that drew further discussion included the M&E plan subcomponent. Here, NASCOP’s efforts in providing technical assistance to better monitor and evaluate KenyaEMR’s progress informed the group that the M&E subcomponent was at a 5-optimized maturity level for the goal status. Since some stakeholders had not known about these efforts when completing their individual assessment, they had initially rated this subcomponent lower. Another example is the KenyaEMR leadership and coordination subcomponent. Workshop discussions revealed that the new DHA’s establishment will be able to play a role in providing an ongoing review of KenyaEMR activities for continuous improvement to meet the system’s strategies. Under the ICT infrastructure domain, reaching consensus on the reliable power/electricity subcomponent took additional time for discussion. This is because ensuring reliable power/electricity at the optimized level is not only within the MoH’s control but also involves other government ministries, such as the Ministry of Energy, which may have different operating schedules. In addition, power reliability varies among different counties, which leads to different experiences from the stakeholders’ perspectives. The business continuity plan (BCP) subcomponent also led to longer discussions as some stakeholders were unaware of an existing BCP for KenyaEMR and the existence of a plan for a digital health strategy.

### Study lessons learned

We learned several important lessons from conducting this assessment:

1. Engaging diverse stakeholder groups to ensure different perspectives and minimize information asymmetry is critical.
2. Adapting to participants’ time constraints is necessary when conducting maturity assessments. For instance, tailored activities to reduce the time burden of participation included converting the toolkit into an online survey for individual self-paced completion of select domains and having all stakeholders complete the ICT infrastructure domain, given they all had relevant expertise, helped facilitate consensus-building work during the workshop.
3. Stakeholder engagement and feedback helped identify additional areas not originally part of the SOCI toolkit but of importance to their context. One example is that data security was not explicitly addressed in the assessment as an essential component of digital health solutions in the HIS SOCI toolkit.
4. Designing and organizing a one-day in-person workshop minimized time commitments and boosted attendance. This was especially helpful as the assessment took place during ongoing maandamano/protests in Nairobi that hindered in-person movement. The one-day workshop required a well-planned agenda, especially around the consensus-building process. All stakeholders working on one domain together at the beginning helped participants know what to expect from other domains they would participate in.
5. Incorporating the Delphi method for agreement with a ≥70% threshold in the first round of voting added rigor and structure to the consensus-building process. Further, it utilized digital and anonymous voting to capture real-time consensus-building in round one and two streamlined consensus-building which saved time during the workshop.
6. The improvement roadmap was a live document that captured action plans collaboratively, accurately, and transparently, helping participants follow along and provide input.
7. Having a plenary at the end of the workshop to present the improvement roadmap facilitated rich input from the participants.
8. Holding one primary session for domain 3 (ICT infrastructure) allowed stakeholders to exercise building consensus before continuing to their respective breakout groups. Domain 3 was the most appropriate domain for a central group discussion as all stakeholders had expertise and experience in this area, and it only contained five subcomponents, making it manageable to go through with all stakeholders.
9. Concurrent breakout sessions after the first consensus-building exercise made it easier to manage time, the consensus-building process, and developing the improvement roadmap.

These lessons will benefit future maturity assessments, including KenyaEMR, other digital health solutions in Kenya, and other countries interested in conducting similar assessments. A future assessment of KenyaEMR will be important, as the system is undergoing a pilot of OpenMRS 3.X upgrade, a facility-wide version that can be used in all departments and points-of-service (not just HIV), in select sites [22, 38].

This assessment provides a baseline to understand and measure KenyaEMR’s progression across different HIS domains, which will be beneficial in tracking maturity progression following future assessments. Additionally, this is the first assessment focused on one digital health information system, in comparison to other maturity assessments that focused on overall national health information systems such as the HIS interoperability assessment done in Kenya [28] and other SSA countries such as Tanzania, Ghana, Uganda, Ethiopia, and Nigeria [5, 26, 27, 33, 39]. This provides a use case example for a single system maturity assessment which can be used for other individual digital health solutions in Kenya and beyond with the appropriate maturity progression measurement scale definition adaptations. It is also an example of adaptations to country contexts, such as online survey options and anonymous voting to assist with the consensus-building process. The engagement of experts from diverse organizations and roles at the national and county levels, donor agencies, and implementing partners, ensured the credibility of the assessment. Additionally, tangible action plans will address identified gaps for the MoH to utilize as the next steps.

### Limitations

While the study included strategies to enhance rigor and promote inclusion, such as incorporating diverse perspectives across the leadership team and assessment stakeholders, several limitations should be considered when interpreting the findings. There may have been some desirability bias in scoring the desired goal status to be at a higher maturity, potentially setting unrealistic expectations for system development within the proposed timeframe. Additionally, despite a rigorous consensus-building process that included a two-round adapted modified Delphi method, anonymous polling, and group discussions, power dynamics or organizational hierarchies among participants could have affected the process, potentially masking other perspectives that might have offered additional insights. Also, the purposive selection of stakeholders based on their expertise on KenyaEMR may have left out other perspectives, such as those in academia. This assessment reflects the data collection timeframe and is a snapshot of where KenyaEMR was at a point in time. Stakeholders indicated that data security was not explicitly addressed in the assessment as an essential component of digital health solutions in the HIS SOCI toolkit.

## Conclusion

The HIS SOCI toolkit used for the KenyaEMR maturity assessment covered a comprehensive range of 39 subcomponents within five core domains. This study’s findings provide valuable information for system improvement and inform strategic planning for digital health initiatives in Kenya and similar settings in LMICs. Study results show that KenyaEMR’s maturity progression is at advanced levels, compared to regional neighbors who conducted a similar assessment of their national HIS. The HIS management and workforce domain scored the lowest, while the HIS standards and interoperability domain scored the highest maturity level. There are promising commitments from the national government to digitize all sectors, which is expected to continue evolving the digital health space in Kenya. This study provides an example of assessing the continuous progress of a single digital health solution, baseline data for future KenyaEMR assessments, and an action plan to assist in closing existing gaps. These are expected to better help KenyaEMR reach the desired goal status set by the end of 2027. Conducting routine maturity assessments with applicable updates to the KenyaEMR adapted maturity measurement scale is essential as the system evolves to reflect system changes. This study incorporated additional assessment elements to ensure the successful completion of the assessment, which can enrich and strengthen the assessment in the future.

## Supplementary Information

Below is the link to the electronic supplementary material.

Supplementary Material 1

## Data Availability

The datasets used and/or analyzed during the current study are available from the corresponding author on reasonable request.

## Abbreviations

BUSPH: Boston University School of Public Health
CDC: U.S. Centers for Disease Control and Prevention
DHA: Digital Health Agency
DHIPR: Directorate of Digital Health, Information, Policy, and Research
EMR: Electronic Medical Records
HIS: Health Information System
SOCI: Stages of Continuous Improvement Toolkit
HIV: Human Immunodeficiency Virus
ICT: Information, Communication, and Technologies
KenyaEMR: Kenya Electronic Medical Record
LMIC: Low-and-middle-income countries
MoH: Ministry of Health
NASCOP: National AIDS and STI Control Programme
OpenMRS: Open Medical Record System
PEPFAR: U.S. Presidents Emergency Plan for Aids Relief
SSA: sub-Saharan Africa
USG: U.S. Government
WHO: World Health Organization
