# Supplementary material for: Maturity assessment of KenyaEMR: advancing digital health solutions through continuous improvement in Kenya
Source: BMC Health Serv Res. 2026 Jun 17;26:1062. doi: 10.1186/s12913-026-14965-6 (PMC13449493; doi:10.1186/s12913-026-14965-6)
Supplement: Supplementary file 1 [file 12913_2026_14965_MOESM1_ESM.docx]

## **Additional file 1:** Maturity Stages Measurement Scale Definitions Adapted for the Assessment of Kenya Electronic Medical Records (KenyaEMR) system from the Health Information System Stages of Continuous Improvement (HIS SOCI) Toolkit

| **HIS Code** | **Subcomponent** | **Definition -KenyaEMR** | **Maturity Stages** | | | | |
| --- | --- | --- | --- | --- | --- | --- | --- |
|  |  |  | **Emerging (1)** | **Repeatable (2)** | **Defined (3)** | **Managed (4)** | **Optimized (5)** |
| **I.** | **HIS LEADERSHIP AND GOVERNANCE** | | | | | | |
| **I.A.** | **HIS strategy** | | | | | | |
| I.A.1. | HIS strategic planning | An HIS strategic plan sets out, in the context of KenyaEMR, a vision for management and use of health information, a plan of action for delivering the vision, and arrangements for M&E. NASCOP and counties may have either an eHealth strategy specific to KenyaEMR, or a broader HIS strategy that includes EMRs. | There is awareness of the need to develop a KenyaEMR and/or EMR strategic plan or update the existing one, but the planning process is at an early stage. Planning is mostly focused on small or short-term projects. | KenyaEMR strategic plans are current and developed by subject matter experts but not vetted with all key stakeholders. Strategic plans are developed by different bodies focusing on specific domains and may not include all relevant HIS activities. | There is an established KenyaEMR strategic planning process that involves key KenyaEMR/EMR stakeholders and is formally approved by the Ministry of Health. There is a current KenyaEMR/ EMR strategic plan that includes standards, and appropriate technical and service delivery aspects, which are developed using a gender-equity lens, as well as ensuring that there are financial and human resources to deliver them. | A budgeted KenyaEMR and/or EMR strategy is aligned with and integrated in the national health plan/strategy and is aligned with the health sector M&E plan. National level implementation is monitored, and there is a set schedule for periodic review and update for corrective action by a designated government led work group. | A continuous improvement planning process is maintained. KenyaEMR/EMR strategic planning is responsive to changing health domain needs/priorities reflected in the health sector plan and the M&E plan. |
| I.A.2. | Monitoring and evaluation (M&E) plan | A framework for regular evaluation (both formative and summative) of KenyaEMR activities and implementations to measure progress on milestones and goals in the delivery of healthcare services and to ensure alignment of KenyaEMR activities with EMR/HIS strategy and the desired impact on service delivery. | There is awareness of the need to monitor and evaluate KenyaEMR activities and implementation to ensure alignment with EMR/HIS strategy and compliance with standards and guidelines. KenyaEMR evaluations are conducted on an ad hoc basis. Tools for monitoring and evaluating KenyaEMR activities and implementation are not harmonized with planning across key stakeholders (i.e., NASCOP, implementing partners, etc.). | An organizational structure is in place to monitor, evaluate, and measure results for major KenyaEMR activities and implementation. Processes to monitor, evaluate, and measure KenyaEMR activities and implementation are project-based or lack institutional support. There are efforts to coordinate and harmonize evaluation tools across implementations of KenyaEMR. | There are defined and standardized processes and tools for evaluating KenyaEMR activities and a relevant governing authority is established. Data collected during evaluations are consistently used to prioritize and align KenyaEMR activities and implementation. | There is ongoing M&E to ensure alignment of KenyaEMR activities with EMR/HIS strategy and desired impact on healthcare service delivery. Performance reports are shared with the relevant KenyaEMR/EMR leadership groups. | Feedback from evaluation is used for continuous improvement of M&E systems, processes, and capabilities for alignment with the current EMR/HIS strategy and best practices. Ongoing impact analysis is based on evaluations to optimize resource allocation and project future investments. |
| **I.B.** | **Policy, legal, and regulatory framework and compliance** | | | | | | |
| I.B.1 | Existence of EMR policies and procedures | Documented KenyaEMR policies and procedures that outline a deliberate system of principles to guide decisions and achieve KenyaEMR outcomes. This framework designates an appropriate entity to oversee adherence to procedures and policies related to data management, data sharing and use, privacy and security, and business process continuity. | Some procedures, regulations, and policies exist, but most are based on general information technology (IT) rather than EMR needs. The need for policies and regulations that govern KenyaEMR has been identified. | Some procedure, policies and regulations (for data management, data sharing and use, privacy and security, and business process continuity) that govern EMR are implemented and are used in managing KenyaEMR activities in selected settings. | Comprehensive KenyaEMR strategies, regulations, and policies are created by a clearly defined body constituted of people/organizations from a wide variety of backgrounds. Core KenyaEMR policies and regulations are being used and monitored following established guidance nationally and/or regionally for managing KenyaEMR activities. | Explicit EMR national procedures, policies addressing data standards and interoperability, privacy and security, information and communication technologies (ICT) infrastructure, data stewardship, and data use agreements are widely available, used, and integrated in KenyaEMR processes and compliance is monitored by the relevant MoH agencies (i.e., TWG, certification committee of agency, etc.) | KenyaEMR procedures and policies are regularly enforced and actively reviewed and updated by a EMR TWG as necessary, to reflect changes within the health domain. |
| I.B.2 | EMR Policy compliance enforcement | Specified mechanisms and regulatory KenyaEMR protocols in line with global and in-country MoH Digital Health standards to ensure adherence to organizational policies (NASCOP and counties), procedures, and best practices related to HIS, including standards for data exchange, messaging, and security. It also means adherence to applicable laws, relevant industry standards, and internal policies (e.g., codes of conduct). | Some processes and procedures for enforcing compliance of KenyaEMR with global and MoH Digital Health standards, policies, and regulation exist, but are mostly based on general IT. There are no structured processes to address noncompliance. | Some processes and procedures for enforcing KenyaEMR compliance exist with global and MoH Digital Health standards but are limited to specific domains or activities. Some processes for remediation of noncompliance issues may exist but are not standard or regular. | A clearly defined body and process and procedures is in place to ensure KenyaEMR compliance with global and MoH Digital Health standards, policies, and regulations. A structured process exists for remediation if noncompliance is identified. | A process to review, validate, and enforce KenyaEMR implementation of global and MoH Digital Health standards policies, and regulations in HIS is followed regularly. Metrics on compliance and noncompliance are collected, recorded, and reported. | Processes and procedures for enforcing KenyaEMR compliance with global and MoH Digital Health standards and policies are actively reviewed and updated, as necessary, to reflect changes within the health domain. |
| **I.C.** | **HIS leadership and governance organizational structures and functions** | | | | | | |
| I.C.1 | KenyaEMR leadership and coordination | The exercise of technical, political, and administrative authority to manage KenyaEMR affairs at all levels of a country’s health system. The governance structure consists of the mechanisms, processes, and institutions through which actors and stakeholders articulate their interests, exercise their rights, meet their obligations, mediate their differences, and oversee the functioning of the KenyaEMR. | Where leadership exists, it is exercised within programs or units at the local level. Programs and units manage KenyaEMR’s based on their independent needs; there is no central oversight or guidance. The KenyaEMR leadership and coordination body's functions are not clearly defined. Meetings happen infrequently or on an ad hoc basis. | There are efforts to formalize KenyaEMR leadership and coordination with a clearly defined scope of work and operational structure. An KenyaEMR leadership and coordination body provides oversight and coordinates management of KenyaEMR activities in selected cases or activities. | A national-level cross-agency coordination group exists to oversee policies, scope of work, roles, and responsibilities for coordinating KenyaEMR activities centrally. This coordination group includes management and subject-matter experts and meets on a regular basis to identify, prioritize, and coordinate KenyaEMR activities. | Coordinated national-level oversight is integrated in the KenyaEMR health strategy as an institutional structure and facilitates the implementation of HIS strategy. The group meets regularly (at least annually, depending on organizational needs), and there is an established process for sharing and reviewing KenyaEMR information with all KenyaEMR stakeholders. | There is ongoing review by the national-level coordination group of KenyaEMR activities, purpose, process, resources, team composition, attention to gender and equity, and communications for continuous improvement to meet changing KenyaEMR strategy and/or health goals. |
| I.C.2 | KenyaEMR organizational structure and functions | Defined KenyaEMR organizational structures and process, including stakeholder matrix (with clear descriptions and responsibilities). There are clear roles for those responsible for development, implementation and monitoring of KenyaEMR. | A stakeholder matrix with defined KenyaEMR roles may exist but is limited to certain organizations. There is no defined or formal process for review, updates, or alignment of the KenyaEMR organizational structure or functions. | A stakeholder matrix with KenyaEMR job titles and corresponding job descriptions exists but there is no formal process for regular updates and alignment. | An established, standardized process is in place for developing and reviewing KenyaEMR job titles, series/cadres, functions, and responsibilities. | A formal process exists for review and updates of KenyaEMR stakeholder matrix (including NASCOP and counties) and job descriptions and is aligned with the HIS/health strategy. There is an established plan for career training and retention for each job series/cadre. | The KenyaEMR stakeholder matrix (including NASCOP and counties) and functions are reviewed regularly for continuous improvement and to ensure alignment with the strategic plan. |
| **II.** | **HIS MANAGEMENT AND WORKFORCE** | | | | | | |
| **II.A.** | **HIS workforce capacity and development** | | | | | | |
| II.A.1 | KenyaEMR workforce competencies (knowledge, skills, and abilities) | Availability of adequate personnel with characteristics, attributes, and capabilities to perform a task/set of tasks to achieve clearly defined results for KenyaEMR development, implementation, and management. | Relevant HIS and/or health informatics competencies for cadres of KenyaEMR users are not defined or documented. The competencies (knowledge, skills, and abilities) required to accomplish tasks exist locally or for specific KenyaEMR activities. | Some KenyaEMR workforce competencies necessary to accomplish KenyaEMR implementation activities are documented and are used to develop clear descriptions of duties and responsibilities for each job cadre in limited facilities. | There is an established and disseminated national EMR organizational chart with clear descriptions of duties and responsibilities for each job cadre. Workforce competencies are aligned with the KenyaEMR strategic plans, as well the organizational (MoH, NASCOP, and counties) mission, vision, values, and business strategy. Informatics and project management concepts are used in developing, implementing, and managing KenyaEMR activities and projects. | KenyaEMR capability assessments and analyses are conducted regularly to explore opportunities for competency improvement. KenyaEMR training program courses are aligned with established core competencies, to meet training needs. There is an established career path for KenyaEMR workforce, which incorporates a strategy for capacity building. | There is an established schedule for reviewing EMR competencies, to ensure continuous KenyaEMR performance improvement and alignment with healthcare goals. |
| II.A.2 | KenyaEMR training and education (includes continual professional development) | An organized activity with clear learning outcomes that aims to impart knowledge and skills, shape attitudes, and develop specific competencies and capabilities in KenyaEMR personnel. | Training and education programs for KenyaEMR competencies are at the initial stages of development. The HIS training curriculum is not standardized. Trainings are conducted on an ad hoc basis, and they mostly focus on specific software. | Training is regarded as critical, and processes to identify relevant courses for different KenyaEMR staff cadres exist in limited settings. HIS-related academic courses using KenyaEMR are available for specific HIS domains or activities. | Training, academic curricula, and processes for developing training and education programs to build KenyaEMR skills and competencies nationally are standardized and include a gender-related component. Clear and measurable learning outcomes are defined for training courses. A designated government authority to oversee training and curricula exists. | Training and education plans are integrated into KenyaEMR implementation plans and the results are measurable. Training and education programs conducted periodically at government-designated institutions to refresh existing skills and impart new skills are reviewed on a regular basis by the designated authority to ensure alignment with HIS needs and technology. There is sufficient infrastructure to support different modes of training, including distance learning. | Regular feedback from systematic curriculum review, training, and piloting innovative ideas are used for KenyaEMR continuous improvement. The training approach is proactive and student-centric, with reusable content driven by the implementation plans and revised to meet emerging health-sector needs. |
| II.A.3 | HR policy (workforce planning, human capacity, and interventions) | A set of principles, guidelines, and norms that guides workforce planning and ensures sufficient workforce capacity and management of staff that support KenyaEMR. | Workforce planning for KenyaEMR is conducted on an ad hoc basis in response to more immediate operational needs in local settings or specific implementation. Data on vacant HIS positions and staffing needs for KenyaEMR are not collected or are collected on paper and must be integrated with other information for planning and allocation manually. Adequate staff exists only in some locations, typically in large cities, and KenyaEMR roles and responsibilities are not well defined. | Workforce planning for KenyaEMR tends to focus on recruitment. Data on KenyaEMR vacant positions are collected and managed using a human resources information system (HRIS) in limited settings, and some KenyaEMR competencies, roles, and responsibilities of staff are clearly documented. | Data on vacant KenyaEMR positions are collected and managed using HRIS nationally. Numbers are sufficient to meet KenyaEMR workforce needs at national but not subnational levels. Reports generated from HRIS are disaggregated by cadre and location for KenyaEMR workforce planning and allocation. Hiring mechanisms distribute staff to some subnational facilities. KenyaEMR competencies, roles, and responsibilities of staff performing KenyaEMR functions are clearly documented and disseminated to the concerned staff. | Data on vacancies and staffing needs for KenyaEMR are collected and managed in the HRIS on a regular basis and used to inform hiring, distribution of staff, and training and education needs, and to advocate for budgets to meet KenyaEMR needs nationally. Staffing needs are met at county levels. Workforce analysis is conducted to forecast future demands. Hiring mechanisms distribute staff to all locations where they are needed. Human capacity needs are integrated in the HIS and/or health plan and monitored by a designated government authority. | Workforce planning, human capacity requirements, and availability are continuously improved for KenyaEMR based on a national HIS strategic plan and focus on long-term interventions in areas such as competency management, employee engagement, skills acquisition, and capability development. |
| **II.B.** | **Financial management** | | | | | | |
| II.B.1 | KenyaEMR financing plan | There are established legal and administrative financial systems and procedures that enable the Ministry of Health, its agencies and organizations to conduct KenyaEMR activities. These procedures are designed to ensure proper and appropriate use of funds, while adhering to defined standards of probity and regularity. Activities include management and expenditure oversight, financial accounting, reporting, and asset management. | Financing and investments are limited to specific projects. The KenyaEMR is not included in the healthcare budget as a separate requirement. | HIS financing and investments are allocated on a case-by-case basis and focus on short-term projects, not long-term strategic priorities. The need for a designated budget for KenyaEMR activities has been recognized but may not yet exist. | Standard processes for qualifying HIS projects for funding and monitoring of expenditures are established and disseminated to all key stakeholders. There is a line item for EMR in the healthcare budget, and KenyaEMR budgeted details are developed for national and/or subnational HIS in the country. EMR is funded through capital financing or alternative financing models, such as public-private partnerships. | There is a multi-year HIS financing strategy aligned with healthcare and HIS strategic priorities, and sources are identified for sustained KenyaEMR activities. KenyaEMR implementation is funded using various types of financing, such as capital financing, revenue, public-private partnerships, and grants. Expenditure reports are shared with the relevant HIS team/unit. Financial audit processes are in place and regularly carried out to promote accountability in HIS spending. | An established HIS financial management system is owned, reviewed, tracked, and revised by the government. HIS investments consider different healthcare priorities and goals and strategically invest in capabilities to support future KenyaEMR initiatives. Financial planning is done for the entire KenyaEMR implementation lifecycle and includes sustainability. |
| II.B.2 | KenyaEMR Resource mobilization | The process for obtaining new and additional financial resources for KenyaEMR by organizations utilizing it (including NASCOP and counties). It also involves making better use of and maximizing existing financial resources. | A resource mobilization plan for KenyaEMR (at NASCOP and counties) activities is under development or occurs in an ad hoc manner. | A resource mobilization plan exists for specific KenyaEMR activities. The need for a standardized plan for HIS resource mobilization plan for EMR activities is recognized and such a plan is under development with NASCOP and counties leadership/involvement. | The resource mobilization plan for EMR including KenyaEMR is documented and disseminated, and progress is approved and monitored by a designated government department/team at the appropriate level of implementation (national, subnational). | The resource mobilization plan for EMR activities is integrated into the HIS and/or health plan at the appropriate level of implementation (NASCOP and counties). Review processes are standard and happen regularly, and findings are shared with relevant stakeholders. | The resource mobilization plan is periodically reviewed/revised to accommodate financial requirements needed to support evolving KenyaEMR activities and emerging health sector needs at the appropriate level of implementation (NASCOP and counties). |
| **III.** | **HIS INFORMATION AND COMMUNICATION TECHNOLOGIES (ICT) INFRASTRUCTURE** | | | | | | |
| **III.A.** | **Operations and maintenance** | | | | | | |
| III.A.1 | Reliable power/  electricity | Sufficient and reliable power supply for relevant offices and health facilities service delivery points to enable use of KenyaEMR for management and use of health (individual, population, and program) data. | Information on electricity/power access, sources and reliability at healthcare facilities, and subnational and national level offices is limited and only collected when planning for specific KenyaEMR activities. Procedures for maintaining power infrastructure exist in limited settings. | Data are collected on electricity/power access, sources, and reliability to support KenyaEMR infrastructure but is not harmonized across healthcare facilities, or relevant offices (i.e., where servers are hosted). Some procedures for maintaining power infrastructure exist. | An established governing body oversees standardized procedures for tracking and maintenance of electricity/power access, sources and reliability supporting HIS infrastructure including KenyaEMR at healthcare facilities and relevant offices. Standardized metrics for tracking power outages and the duration of outage are defined. | Electricity/power access, sources, and reliability profiles for healthcare facilities and relevant offices using or responsible for KenyaEMR are reviewed and updated on a regular schedule. The power infrastructure is monitored on a regular basis by the governing authority to ensure reliability at all levels. Data from electricity profiles is used to develop innovative solutions to improve access and reliability of electricity. | Information collected on the electricity access, sources, and reliability profile is used for planning and continuous improvement of KenyaEMR implementation and to meet emerging needs of the health sector. |
| III.A.2 | ICT business Infrastructure support and maintenance | Design and planning, operations management, and technical support for ICT infrastructure maintenance used for KenyaEMR. | There is basic support for ICT or electronic systems equipment installation and maintenance, but it is not standardized or dedicated to infrastructure for KenyaEMR. Maintenance for the network and hardware is a mix of reactive and ad hoc preventive maintenance. | There is a recognized need to standardize processes to oversee and support ICT infrastructure, but no established or harmonized process exists specific to HIS/KenyaEMR need. | There is adequate ICT capacity to support ICT equipment installation and maintenance including KenyaEMR. There is formal technical assistance to build a system for ongoing technical support and maintenance. Standard operating procedures (SOPs) exist that detail the procedures for routine network and hardware maintenance supporting KenyaEMR. | Operations and maintenance services are included in the HIS/KenyaEMR plan or health plan. Operations and maintenance SOPs are implemented consistently. Data is collected and regularly reviewed on the ICT infrastructure operations and maintenance as mandated by the HIS/KenyaEMR plan or health plan. A functional and always-staffed help desk exists at national and subnational levels. | HIS ICT equipment maintenance and user support are integrated in an EMR strategic plan, which includes financing and technical support for continuous improvement of ICT infrastructure and support. An operations and maintenance services mechanism are continuously reviewed and adapted with evolving HIS/KenyaEMR requirements and following industry-based standards. |
| III.A.3 | Computing devices/ Hardware | Computing devices and hardware, including servers and virtual private networks (VPNs), which forms the foundation upon which HIS activities for KenyaEMR and health information exchange across services, geographic and health sector boundaries are adequate and implemented. | Service delivery points and relevant offices using and managing KenyaEMR has few computers to support it or hardware is dedicated to specific HIS activities. No guidance exists on the minimum specifications for the country's hardware for the health sector. | An audit report shows the required KenyaEMR computing devices/hardware specifications for health facilities, and relevant offices at national and subnational levels. Fewer than half relevant offices have adequate hardware (e.g., computers, printers, connecting devices). | About half or more of the health facilities and relevant offices at the national and subnational levels have adequate computing hardware, including backup services to support KenyaEMR. A plan exists for backup and recovery as well as a plan for replacing old and broken computing devices. | Most of the health ministry's national and subnational offices have adequate hardware. The computing and network hardware is working optimally to support KenyaEMR operations. | All or most of the service delivery points and relevant offices at the national and subnational offices using or supporting KenyaEMR have adequate computing hardware for KenyaEMR. Hardware is monitored and evaluated on a regular basis to ensure supported functions are operational. |
| **III.B.** | **Communication network (LAN and WAN)** | | | | | | |
| III.B.1 | Networks and Internet connectivity | Reliable and secure connectivity using necessary components such as routers, LAN, WAN, VPNs and gateways for KenyaEMR to connect and exchange data appropriately in the health domain. | Network and Internet connectivity infrastructure exists only at the national level and some subnational level offices. No stable network connection exists at subnational levels or health facility level to support KenyaEMR. | A national plan for network management to ensure high up-time is in place. Adequate dedicated network and Internet connectivity are in place to meet the basic needs of health data management and support normal functioning of KenyaEMR in the relevant offices at all levels. | A national plan for network management to ensure high up-time is in place, with clear procedures to follow in case of network failure, to ensure high up-time. Assessments are conducted at least annually by a designated governing body to identify gaps in ICT infrastructure supporting KenyaEMR. | Most counties have a working computer network connection and about half of health facilities have a strong and reliable network connection to support KenyaEMR. A dedicated network support team is in place. Gaps in connectivity are documented and addressed in standard process. | All relevant services points and offices where KenyaEMR is used have reliable network connections or mechanisms that support operation in offline mode (and syncing later on). A team with adequate financial, human, and technology resources is dedicated to support connectivity. Industry-based standards are followed to manage and maintain KenyaEMR. |
| **III.C.** | **Business continuity** | | | | | | |
| III.C.1 | Business continuity processes and policies | Business continuity is about devising plans and strategies that enable KenyaEMR and its respective products to continue business operations and enable it to recover quickly and effectively from any type of disruption, whatever its size or cause. | An HIS business continuity plan (BCP) is under development or may exist only for specific KenyaEMR activities. | A BCP exists and is utilized for KenyaEMR but is not harmonized across HIS health-sector needs. Efforts to document or standardize a BCP may exist at the national level or to address national-level needs. | KenyaEMR utilized a BCP that is documented, published, monitored, and audited by a government- led working group, to ensure compliance and evaluate performance against metrics outlined in the BCP. | A BCP is integrated in the HIS strategic plan and regularly managed by the government-led working group to address goals and gaps in meeting HIS needs, including KenyaEMR. | A BCP is reviewed and revised periodically including input from KenyaEMR implementation to ensure that it meets the evolving HIS and health information priorities. |
| **IV.** | **HIS STANDARDS AND INTEROPERABILITY** | | | | | | |
| **IV.A.** | **Standards and guidelines** | | | | | | |
| VI.A.1 | HIS standard guidelines | KenyaEMR adherence to EMR standards and guidelines to enable consistent and accurate definition, collection, and exchange of health information across HIS and services. | Some KenyaEMR features and functions adhere to limited parts of the EMR standard guidelines and are in use, but they are defined locally or for specific programs. KenyaEMR stakeholders may see little value in the use of guidelines, because of cost and difficulty in adopting them. | Some KenyaEMR features and functions adhere to EMR standard guidelines and are used for KenyaEMR activities. There is awareness of the key role that EMR standard guidelines play in supporting KenyaEMR and healthcare operations. | Formal processes and procedures for creating, adopting, and managing for KenyaEMR to adhere to EMR standard guidelines are defined and disseminated. A formal body/group and clearly defined processes are in place for ensuring KenyaEMR adherence to EMR standard guidelines, and resources are allocated for the activities. | The existing KenyaEMR aligns with the practice, workflows, and business needs, defined in the up-to-date EMR stand guidelines. There is a plan for regular review and updates the EMR standard guidelines are recognized by key stakeholders and widely adopted for KenyaEMR activities and implementations. | The process of creation, adoption, and management of EMR standard guidelines is continuously improved from lessons learned and experiences of KenyaEMR implementation. KenyaEMR practice, workflows, and business needs inform creation or adoption of new EMR standard guidelines. |
| IV.A.2 | Data set definitions (clinical, laboratory, commodities, and indicator) | Common data sets, such as those defining clinical care, surveillance, and/or M&E programs, are defined along with distinct data elements. | Some data sets for clinical care and monitoring and evaluation (M&E) exist for KenyaEMR for a specific program. | Some data sets for managing clinical care, surveillance, and M&E for KenyaEMR exist but are used for limited programs. Surveillance data sets may exist for programs/diseases that previously had an outbreak. There are efforts to harmonize data sets with those from international organizations and standards. | There is a formal, recognized body and defined guidelines for creating, updating, and maintaining national health data sets used in KenyaEMR (including sex disaggregation, where applicable). Core minimum data sets, including those for clinical care, surveillance, laboratory, pharmacy, or M&E programs, are defined and used nationally and regionally. Data sets for KenyaEMR are harmonized with those from international organizations and recognized standards. | All data sets (including sex disaggregation, where applicable) are developed in line with national guidelines and used in KenyaEMR.  The national indicator set is integrated in the national health plan or strategy, and implementation is reviewed by the designated unit or department. There is a plan for regular reviews and updates of datasets in KenyaEMR. | The national data sets (including sex disaggregation, where applicable) are managed in a central system with capability to publish and KenyaEMR subscribe to it. KenyaEMR information needs, and a corresponding indicator set are periodically reviewed, to align with evolving HIS and health goals. |
| IV.A.3 | Data and exchange standards | KenyaEMR utilizes a data and exchange standards l for organizing electronic information in a common format, such as XML and HL7, to facilitate linking and sharing of data with other systems (e.g., databases, applications, etc.). | KenyaEMR is not using defined technical standards in the country for data management and exchange or may exist for specific diseases or HIS activities. | The country has adopted and/or developed standards for health data management and exchange, but standards used in KenyaEMR may be localized to specific projects. There is an effort nationally to standardize the process. | Standards for health data management and exchange are approved and incorporated in KenyaEMR. An interoperability laboratory exists for testing KenyaEMR data exchange with other systems and for certification purposes. | The national data management and exchange standards are integrated in the national HIS and/or health plan are implemented in KenyaEMR. Exchange standards in KenyaEMR are tracked, monitored, and reviewed through a standardized process. | Electronic data transmission is the default method for sending and receiving data between KenyaEMR and other information systems at service delivery points and subnation and national levels. Industry-based data sharing standards are followed. |
| **IV.B** | **HIS core services** | | | | | | |
| IV.B.1 | Master facility list | KenyaEMR utilizes the master facility list (MFL) established by MoH in the country and comprises administrative information and information that identifies each facility (signature domain). | A facility list exists only for specific projects or programs that are utilized by KenyaEMR. | There is no one government-developed facility list. Existing facility lists are not harmonized and KenyaEMR relies on partner/funder supported lists. The health ministry is moving creating a central MFL or health facility registry that can be utilized by KenyaEMR. | A central MFL under the health ministry is being used by KenyaEMR. There is a defined guideline for managing and updating the list, overseen by a designated governing body. Lists are available and shared with stakeholders. | The MFL is updated regularly, to add to it or otherwise revise it. MFL is used by KenyaEMR, and periodic updates are integrated in the HIS plan or health strategic plan. There is an established feedback process to review and address gaps in MFL data/metrics following utilization of KenyaEMR. | The full, coordinated MFL is available as a common and managed registry. The master facility registry has the capability to publish and KenyaEMR subscribes to it. The health ministry (or governing body) regularly reviews and updates the central MFL and shares it with the stakeholder’s following utilization of KenyaEMR. |
| IV.B.2 | Indicator registry | A list of core health indicators that help track progress are managed in an indicator registry that is utilized by Kenya EMR to access indicator definitions and codes. | There is limited understanding of the need to centrally manage indicator metadata in use for different program areas. Metadata are inconsistently collected and rarely consolidated outside of KenyaEMR project artifacts. | Templates are adopted to provide some consistency in content and format of captured KenyaEMR indicator metadata. Metadata are consolidated and available from a single portal for activities and implementation in limited KenyaEMR settings. | The collection of indicator metadata on structured content is established, and scheduled automated extracts are performed for KenyaEMR. Processes for collecting and storing. Indicator metadata for include appropriate disaggregation, including by sex. There is a defined process for updating indicator metadata for KenyaEMR. | A nationally managed indicator registry is the primary location for all indicator metadata for KenyaEMR. Indicator Metadata can be automatically shared with other systems. | There is a nationally managed indicator metadata solution that publishes indicators and KenyaEMR subscribes to it. |
| IV.B.3 | Terminology management | The process through which clinical terminology is collected, managed, and stored in KenyaEMR. | There is limited understanding of clinical terminologies and classification of how they can be used in KenyaEMR. Terminology is rarely consolidated outside of KenyaEMR implementation or project artifacts. | Some EMR products including KenyaEMR use standardized templates to maintain consistency in the content and format of terminology. Metadata utilized by KenyaEMR are consolidated and available from a single portal. | Terminology for a clinical minimum data set is defined nationally and in use for KenyaEMR. There is a defined process for extending or localizing the content of terminologies to meet local needs in KenyaEMR. | A centralized metadata store/terminology service is the primary location for all institutional metadata utilized by KenyaEMR. The existing terminology datastore/service utilized by KenyaEMR is available in human and machine-readable format from a central location. | A metadata solution/service exists and provides a single point of access to published terminology resources and KenyaEMR subscribes to it. Feedback from KenyaEMR implementations is used to improve use of terminologies locally, by extending and localizing the content. |
| IV.B.4 | Unique person identity management | KenyaEMR has the capability to utilize or assign identifiers to uniquely identify persons receiving or providing health services. | There is a basic understanding of the need for unique person identification for KenyaEMR. Use of unique person identification in KenyaEMR is limited to a program or local setting. | KenyaEMR is able to utilize and share unique identifiers developed and assigned by other programs. | KenyaEMR is able to utilize Unique person identifiers that can be used across facilities and services are implemented and are used nationally. | Unique person identification is a core HIS/Kenya service that includes the ability to use multiple identifiers or other person data to share information and it’s implemented in KenyaEMR. KenyaEMR uses an established process for assigning unique person identifiers and for unique person identification. | KenyaEMR has the capability to subscribe to a client/patient identity management registry to validate or utilize assigned unique identifiers. Assignment of unique identifiers is integrated in the planning process of new initiatives that KenyaEMR needs to support (e.g., a new health condition not previously captured in KenyaEMR). |
| IV.B.5 | Enterprise architecture | Enterprise architecture (EA) is a method and an organizing principle that aligns functional business objectives and strategies with an information technology (IT) strategy and execution plan. When a country uses an EA to organize its health information system, it is a national EA. | A national health information architecture is nonexistent or not clearly defined. | The government authority has published a national health information architecture document, but KenyaEMR has not developed or harmonized with it. | KenyaEMR contains foundational interoperability tools required to perform its functions with a national health information architecture that is up-to-date and being implemented. | KenyaEMR is integrated with the national health information enterprise architecture or health plan. | Continuous learning, innovation, and quality control exist in the work on HIS/KenyaEMR interoperability and information sharing. |
| **IV.C.** | **Interoperability (data exchange)** | | | | | | |
| IV.C.1 | Person data exchange | Standards-based patient level data exchange between KenyaEMR and other systems. | There is limited capability to support standardized patient data exchange between systems within local or specific KenyaEMR implementation. Data exchange is based on peer-to-peer interfaces, which require major efforts to implement and manual intervention during data exchange. | Capabilities to support automated patient data exchange using internationally recognized standards between KenyaEMR systems exist in limited settings. Major efforts are required to ensure that data exchange standards are integrated in the systems. | There are nationally defined data standards in use based on international standards to support patient-level KenyaEMR data exchange. Most applications have integrated data standards with well-developed interoperability verification regimes. Moderate implementation effort is needed to realize data exchange. | Minimal effort is required to realize KenyaEMR data exchange. Applications are implemented mostly on plan with requisite interoperability quality. Most KenyaEMR components conform to adopted or de facto standards and are certified conformant and interoperable. | KenyaEMR Applications do not require specialized engineering efforts or expertise to implement interoperability. All data exchanges are based on certified interoperability standards. Robust maintenance and update processes are planned and include feedback to standard development organization(s) to improve the standards. |
| IV.C.2 | Aggregate data exchange | Standards-based aggregate data exchange from KenyaEMR | Limited capability exists for exchanging aggregate KenyaEMR data using standards between systems. Aggregate KenyaEMR data reporting is achieved via manual import/export process. | Capabilities to support automated aggregate data reporting from KenyaEMR exist in limited settings. Data formats used for aggregate data exchange are application/software-specific and require major efforts to implement. | KenyaEMR uses the nationally defined aggregate data exchange standards and guides to support aggregate data reporting. Moderate effort is needed to implement automated aggregate data reporting from KenyaEMR at all levels. Processes are in place within KenyaEMR to maintain disaggregated data, such as sex and age, where applicable. | KenyaEMR Applications have the ability to automatically exchange aggregate data, using defined standards at all levels. | KenyaEMR Applications do not require specialized engineering efforts or expertise to implement interoperability. All data exchanges are based on internationally recognized interoperability standards. maintenance and update processes are planned for KenyaEMR, including feedback to standards development organizations (SDO) to improve the standards. |
| IV.C.3 | Commodity management data exchange | Standards-based exchange of data on such healthcare commodities as medical supplies and medications is supported in KenyaEMR. | Commodity data are shared on an ad hoc basis or as needed in KenyaEMR. The data are exchanged using electronic templates in spreadsheet or Word documents via email or file sharing. Electronic data exchange capability for commodity data is limited or is mainly paper-based. | Basic electronic exchange of commodity data occurs from KenyaEMR in limited settings. Data exchange of commodity data is achieved on a peer-to-peer basis between KenyaEMR and other systems, but it is not implemented across the entire supply chain. | There are nationally defined processes and standardized message standards for exchanging commodity data that are utilized in KenyaEMR. | KenyaEMR has capability to exchange data with commodity management and health information systems, such as pharmacy information systems, to support order requests and fulfilment. | KenyaEMR applications do not require specialized engineering efforts or expertise to commodity data exchange. |
| IV.C.4 | Data exchange security | KenyaEMR adherence to organizational and in-country policies, procedures, and best practices related to digital health, including standards for data exchange, messaging, and security. It also means adherence to applicable laws, relevant industry standards, and internal policies (e.g., codes of conduct). | No electronic data exchange conducted using KenyaEMR | KenyaEMR Data exchange is manual, and security standards exists locally or for specific implementations. | Security standards are used within KenyaEMR in limited settings for basic/simple electronic data exchange. Data security depend on the infrastructure and KenyaEMR security in place. | KenyaEMR utilizes Security requirements for data, applications, and network infrastructure to support data exchange are defined and used nationally. | KenyaEMR Data exchange security is reviewed on a regular schedule to ensure privacy, confidentiality, and compliance with set guidelines. |
| **V.** | **HIS DATA QUALITY AND USE** | | | | | | |
| **V.A.** | **Data quality assurance** | | | | | | |
| V.A.1 | Data quality assurance and quality control | Efforts or activities aimed at ensuring that reported KenyaEMR data and results accurately reflect actual health system outputs. This includes data quality assessments and data quality assurance practices. | Procedures for KenyaEMR data collection, processing, analysis, and use exist locally or for specific implementations. Data quality reviews and audits are conducted on an ad hoc basis and are driven by specific data needs. A data-quality assurance plan and national coordinating body (can be a subgroup under the health leadership group/team) to oversee data quality are being discussed, including KenyaEMR. | Procedures used during the course of KenyaEMR data collection, processing, analysis, and use exist in limited settings. Some electronic tools are used to facilitate data quality review and audit process. The document setting forth the data-quality assurance plan is available, and it’s applied in KenyaEMR. | Procedures for KenyaEMR data collection, processing, analysis, and use are defined and implemented nationally (including sex-disaggregated data, where applicable). A regular schedule is defined for conducting KenyaEMR data quality reviews and audits, which include a remediation process to address identified issues. There are procedures for documenting metadata. A national coordinating body to oversee data quality is established and meets regularly (at least annually, will vary with context), and provide guidance applied for KenyaEMR. | Data reviews and audits (including sex-disaggregated data, where applicable) are integrated in the health plans, and are conducted on KenyaEMR on a regular schedule using automated and manual processes to ensure defined levels of quality. Regular meetings on KenyaEMR data quality occur, and issues identified are addressed through an established remediation process, which includes documentation of changes made. Data and metrics on data quality assurance (DQA) are shared with stakeholders. Standards for exchanging data between systems to avoid manual reentry where possible are in use nationally. | There is continuous review and auditing of KenyaEMR through automated and manual processes, to ensure defined levels of data quality. Metrics reported on data quality issues are used for continuous improvement of KenyaEMR. The data-quality assurance plan is reviewed periodically by a national coordinating body and appropriate stakeholders, including KenyaEMR, and the plan is revised to meet evolving data quality needs (including sex-disaggregated data, where applicable). |
| V.A.2 | Data management | The existence and implementation of such processes as standard operating procedures for KenyaEMR data collection, data management guidance, development and production of data collection tools, and supportive supervision/assurance procedures. | KenyaEMR Data management processes are not documented, or documented processes lack clarity and are specific to select HIS activities or projects. | KenyaEMR Data management processes are clearly documented in a nationally recognized and established mechanism to ensure quality of data being transmitted across health system levels. | KenyaEMR Data management processes are up-to-date, implemented, and monitored for compliance, including quality of data transmitted across health system levels. | Standard operating procedures for KenyaEMR national health data management are integrated in the national HIS/KenyaEMR and/or health plans and data quality is actively monitored and shared with stakeholders. | Standard operating procedures for KenyaEMR health data management are periodically reviewed and/or revised to ensure alignment with evolving HIS and/or health data needs. |
| **V.B.** | **Data use** | | | | | | |
| V.B.1 | Data use availability strategy | Data use strategy based on information needs that includes, procedures and policies for data reporting, data dissemination, and production of targeted information products in KenyaEMR for use in decision making and health sector planning. | A KenyaEMR data use strategy document (which includes information needs, data collection and reporting, data dissemination, and production of targeted information products) is absent or briefly outlined. | The data use strategy is documented and available but may not be up-to-date or harmonized across the KenyaEMR implementations. | Implementation of the data use strategy in KenyaEMR (including use of sex-disaggregated data, where applicable) is monitored, reviewed, and overseen by an established governing body for data review. Data is shared with stakeholders. | The data use strategy is integrated in the HIS strategic plan and/or health plans and informs data use strategy in KenyaEMR. Goals and monitoring requirements are determined by the data use strategy and are regularly monitored in KenyaEMR. | The data use strategy is adapted to meet emerging decision-making needs of program managers, policymakers, and providers interacting with KenyaEMR, and it is integrated in the long-term health plan to promote a culture of data use. |
| V.B.2 | Information/  data availability | The process through which data are made accessible and through which KenyaEMR is able to support analysis and use of data to support decision making. | Data collection tools in KenyaEMR are not standard, and procedures are not consistently followed; data are collected and stored in an unstructured format. KenyaEMR design supports availability of data for some users. Each program that utilizes KenyaEMR manages its data separately, and sharing is on ad hoc basis via email, or printed copies. | KenyaEMR is designed and implemented to support longitudinal availability of health data (clinical, surveillance, M&E) in limited settings. The data is available for centrally mandated reporting. Standard operating procedures cover multiple contingencies and are consistently followed. | Most KenyaEMR implementations ensure data availability at all levels for decision support and M&E for authorized users. | The KenyaEMR ensures reliable and appropriate access to data at all levels for authorized users. Changes in KenyaEMR reporting requirements are accommodated with minimal disruptions to data availability. KenyaEMR supports secondary use of data. | Data availability in KenyaEMR is monitored for continuous improvements and to meet emerging health sector needs. |
| V.B.3 | Data use competencies | A measurable pattern of KenyaEMR knowledge, skills, abilities, behaviors, and other characteristics that a person needs to perform work roles or occupational functions related to the use of data, for clinical decision support and public health actioning. | KenyaEMR Data use competencies are unknown, limited to specific instances of HIS implementation, or barely documented. | KenyaEMR Data use competencies are clearly documented and available but may not be harmonized. | KenyaEMR Data use competencies are up-to-date and integrated in training courses/programs (existing and/or new) imparted to users. KenyaEMR Data use competency development is tracked by user type and health system level. | Training programs/courses imparting KenyaEMR data use competencies are integrated in the HIS plan and/or health plans. There is a standardized plan for tracking and measuring competencies and an established feedback mechanism for KenyaEMR users to make updates and address gaps. | KenyaEMR Data use competencies and related training programs/courses are revised to meet emerging and future needs of users. |
| V.B.4 | User/stakeholder engagement | Guidance (methods, processes, and practices) for user engagement to support KenyaEMR progress review, performance monitoring, and assessments. | Guidance (methods, processes, and practices) for user engagement to support KenyaEMR progress review, performance monitoring, and assessment is missing or is in the early stages of discussion. | Guidance (methods, processes, and practices) for user engagement to support KenyaEMR progress review, performance monitoring, and assessment is documented and available. | Guidance for user engagement to support KenyaEMR is implemented, up-to-date, and monitored for compliance by an established governing body. | Guidance for user engagement is integrated in the HIS plan and/or health plans and is utilized for KenyaEMR. Results from KenyaEMR user engagement reviews are incorporated in decision making, including resource allocation and financial disbursement. | Guidance for user engagement to support KenyaEMR is periodically reviewed and revised to address emerging and future decision-making needs of users. |
| V.B.5 | Data synthesis and communication | Guidance outlining the purpose, type, and intended result of EMR information products and dissemination/feedback plan exist and are used in KenyaEMR. | Guidance on the design and use of EMR information products, including KenyaEMR, are not explicitly documented. | Guidance on the design and use of EMR information products, including in KenyaEMR, highlights health/gender equity and is documented and available. | Guidance on the design and use of EMR, information products, including in KenyaEMR, is up-to-date and implemented. | Guidance on the design and use of EMR information products, including in KenyaEMR, with a dissemination and feedback plan is integrated in the HIS plan and/or health plan and monitored for compliance. | Guidance on the design and use of KenyaEMR information products is periodically reviewed and revised to ensure its applicability and relevance to emerging and future decision-making needs. |
| V.B.6 | Reporting and analytics features | Established processes and guidelines to analyze and report against priority health indicators, include HIV, are implemented and applied in KenyaEMR. | Reporting and analytics features are limited in KenyaEMR. Routine reporting of critical health indicators is not standardized across KenyaEMR implementations, and it occurs on an irregular basis. The KenyaEMR reporting process is predominantly manual. More effort is required, because of the large number of tools needed. Reporting or analysis is based on individual or program efforts. | Some efficiency measures have been implemented in the reporting process by consolidating reporting tools in KenyaEMR. Electronic submission of routine KenyaEMR reports from subnational levels exists in limited settings. Analytical features for KenyaEMR reports are in separate statistical software applications. | There are established national processes and guidelines to support standardized routine reporting (including sex-disaggregated data, where applicable) that are applicable to KenyaEMR. Automated data reporting from KenyaEMR to national systems has been implemented in limited settings. Basic reporting and analysis features are available within KenyaEMR and meet the needs of the users at all levels. | Automated data reporting (including sex-disaggregated data, where applicable) is implemented nationally from KenyaEMR on a routine basis. Features to support data discovery, integration, analysis, and visualization exist in KenyaEMR for all levels (health facility, sub-national, national). | Metrics on reporting and analysis capabilities with feedback from users are used for continuous improvement in KenyaEMR. |
| V.B.7 | Data use impact | Clearly defined impact measurement parameters for data use exist and are used in KenyaEMR. | Discussion on the impact of data use has been initiated and measurement parameters may exist for specific KenyaEMR implementation. | KenyaEMR parameters measuring data use impact are defined and documented for specific programs (including use of sex-disaggregated data). | KenyaEMR utilizes parameters for measuring data use impact that are nationally defined, up-to-date, monitored, and reviewed on a regular basis. | KenyaEMR utilizes parameters for measuring data use impact that are integrated in the HIS and/or health plans and plans for process feedback are documented and disseminated. | Evidence of the impact of data use in KenyaEMR is available and guides program adaptation/changes to meet emerging and future health program goals. |
| V.B.8 | Data collection alignment with workflow | The extent to which data KenyaEMR collection processes and tools are aligned with the existing workflows for supporting healthcare providers and program personnel at the different health levels (i.e. national, county, sub-county, health facilities, etc.). | There is limited alignment between KenyaEMR data collection process and the existing workflow. KenyaEMR Data collection is mainly driven by mandated reporting requirements, which may not align with the existing workflow. There is some alignment with data collection and the existing workflow locally or in specific KenyaEMR implementations. Data for existing KenyaEMR data elements are often collected multiple times, through standalone forms or applications and re-entered manually, even within the same workflow. | Some KenyaEMR healthcare-related workflows are documented and are aligned with data collection processes. Some capability to reuse collected data within KenyaEMR for documented workflows exists locally or for specific implementation. KenyaEMR and other HIS applications do not link directly to enable basic data exchange within a workflow. | Nationally defined data collection processes and tools exist in KenyaEMR and align with workflow at all levels. KenyaEMR implementations are customized and implemented, based on established workflows. KenyaEMR and other applications from different entities that serve a common goal are linked and exchange data within a workflow. | KenyaEMR uses standard data collection tools aligned with existing workflow at all levels. Procedures and training materials for data collection using KenyaEMR are developed and updated, based on the existing workflow. Capabilities exist in KenyaEMR to reuse collected data and exchange data seamlessly within the workflows. | Alignment of KenyaEMR data collection processes and tools with the existing workflows are evaluated on a regular basis for continuous improvement. KenyaEMR implementations are exchanging data electronically, according to government-approved standards, and are producing data to meet user needs along the care workflow. |
| V.B.9 | Decision support (clinical or other) | Decision support (DS) provides program and resource managers, clinicians, staff, patients, or other people with knowledge and program-specific and/or person-specific information, intelligently filtered or presented at appropriate times, to enhance health and healthcare. DS encompasses a variety of tools to enhance decision making in the program and/or clinical workflow. These tools include computerized alerts and reminders to managers, care providers, and patients; clinical guidelines; condition-specific order sets; focused patient data reports and summaries; documentation templates; diagnostic support; and contextually relevant reference information, among other tools. | Decision support (DS) in KenyaEMR is focused only on program, resources and/or patient data reports and summaries. Some DS capabilities exist in KenyaEMR locally or for specific implementations. | DS in KenyaEMR exists in some settings and is based on alerts and reminders to the program manager, care provider, and patients. There is a recognized need to establish standard procedures to support decision making that are applicable for KenyaEMR. | DS in KenyaEMR incorporate program and clinical guidelines with defined condition-specific order sets and documentation templates. Knowledge-based engines exist within or are implemented in KenyaEMR in some settings to support decision making. | DS in KenyaEMR is automated, to use the knowledge base for contextually relevant reference information. Decision support capability exists for diagnostic support within KenyaEMR. Assessments to ensure the knowledge relevance, value, and accuracy of algorithms are conducted in KenyaEMR on a regular schedule. | The capability for predictive and retrospective analytics that combine multiple factors, using statistical and/or artificial intelligence techniques, exists in KenyaEMR. There is ongoing review of the knowledge relevance, value, and accuracy of KenyaEMR DS algorithms for continuous improvement. |
